# Facile synthesis of iron nanoparticles from *Camellia Sinensis* leaves catalysed for biodiesel synthesis from *Azolla filiculoides*

**DOI:** 10.1038/s41598-024-61113-3

**Published:** 2024-06-04

**Authors:** Sathish Sundararaman, M. Karthikeyan, J. Aravind kumar, Prabu Deivasigamani, Michael Rahul Soosai, A. Ramaraja, S. Sahana, Badr M. Thamer, Mohamed H. El-Newehy, M. Rajasimman, Praveenkumar T R

**Affiliations:** 1 Department of Chemical Engineering, Sathyabama Institute of Science and Technology, Chennai, India; 2 Department of Energy and Environmental Engineering, Saveetha School of Engineering, SIMATS, Saveetha University, Chennai, 602105 Tamilnadu India; 3 Department of Chemistry, College of Science, King Saud University, P.O. Box 2455, 11451 Riyadh, Saudi Arabia; 4 Department of Chemical Engineering, Annamalai University, Annamalai Nagar-608002, Chidambaram, India; 5 Department of Civil Engineering, Graphic Era Deemed to be University, Dehradun, India; 6 Department of Construction Technology and Management, Wollega University, Nekemte, Ethiopia

**Keywords:** *Camellia sinensis* leaves, Biodiesel, Transesterification, Kinetics, Optimization, Nanobiotechnology, Nanoscale materials, Other nanotechnology, Biological techniques, Nanoscience and technology

## Abstract

Recent years have seen an increase in research on biodiesel, an environmentally benign and renewable fuel alternative for traditional fossil fuels. Biodiesel might become more cost-effective and competitive with diesel if a solid heterogeneous catalyst is used in its production. One way to make biodiesel more affordable and competitive with diesel is to employ a solid heterogeneous catalyst in its manufacturing. Based on X-ray diffraction (XRD) and Fourier Transform infrared spectroscopy (FTIR), the researchers in this study proved their hypothesis that iron oxide core–shell nanoparticles were generated during the green synthesis of iron-based nanoparticles (FeNPs) from Camellia Sinensis leaves. The fabrication of spherical iron nanoparticles was successfully confirmed using scanning electron microscopy (SEM). As a heterogeneous catalyst, the synthesised catalyst has shown potential in facilitating the conversion of algae oil into biodiesel. With the optimal parameters (0.5 weight percent catalytic load, 1:6 oil—methanol ratio, 60 °C reaction temperature, and 1 h and 30 min reaction duration), a 93.33% yield was attained. This may be due to its acid–base property, chemical stability, stronger metal support interaction. Furthermore, the catalyst was employed for transesterification reactions five times after regeneration with n-hexane washing followed by calcination at 650 °C for 3 h.

## Introduction

The advancement of sustainable energy sources is crucial because of the rising energy requirements and the exhaustion of non-renewable resources. Fossil fuels are indispensable to the global economy as they are crucial for the manufacturing of plastics, fertilisers, and the energy required for lighting, heating, and transportation. The demand for fossil fuels rises in tandem with population growth and increased economic output. Studies suggest that when a country’s GDP per capita rises, there is a corresponding increase in the demand for fossil fuels. This, in turn, results in heightened rivalry for these finite resources. Furthermore, the concentration of carbon dioxide (CO_2_) in the atmosphere is increasing as a result of the combustion of fossil fuels^1^. This might potentially result in severe climate change, with global consequences of a disastrous kind. Petroleum remains a limited resource, and eventually, its extraction will become prohibitively costly or maybe unfeasible. In light of these factors, endeavours are on to cultivate sustainable energy alternatives capable of substituting fossil fuels, enhancing fuel accessibility for all nations, and significantly mitigating carbon emissions. We can reduce our dependence on fossil fuels by integrating many methods, even though it is unlikely that any single option will be perfect. Various technologies have been explored as potential sources of sustainable energy. The primary task that remains is to establish sustainable energy industries that can effectively rival conventional energy sources in terms of cost. Electric power is produced by the utilisation of both liquid fuels and fossil fuels. Nuclear power, solar power, hydroelectric, geothermal, and wind power are some of the several technologies that have the potential to provide energy while minimising air pollution. Nevertheless, research on sustainable alternatives to liquid fossil fuels is still in its early stages. Generally, the phrase “biofuels” refers to the most favourable choices for the future^2^. This phrase refers to a wide range of technology that may be utilised to generate energy through various biological processes. The primary biofuel production methods now employed include the use of land-based plants, which are then converted into ethanol. This conversion can occur through procedures such as converting maize starch into sugar and then into ethanol, or converting sugarcane sugars directly into ethanol. An illustration of a successful regional plan is the transformation of sugarcane into ethanol in Brazil. Soybean and palm kernel oils, among other oils, are utilised to a lesser extent in the production of biodiesel^3^. Although these approaches are effective on a local level, it is evident that they are not sustainable due to the significant amount of arable land required to replace a large quantity of petroleum with them. Numerous hybrid techniques are available, either proposed or currently employed, such as gasifying biomass leftovers to provide syngas for the production of liquid fuels, and fermenting extracted cellulose sugars into fuel. Although petrol may be manufactured through several means, these technologies alone cannot meet the global need for liquid fuels^4^.

**Figure 1:**
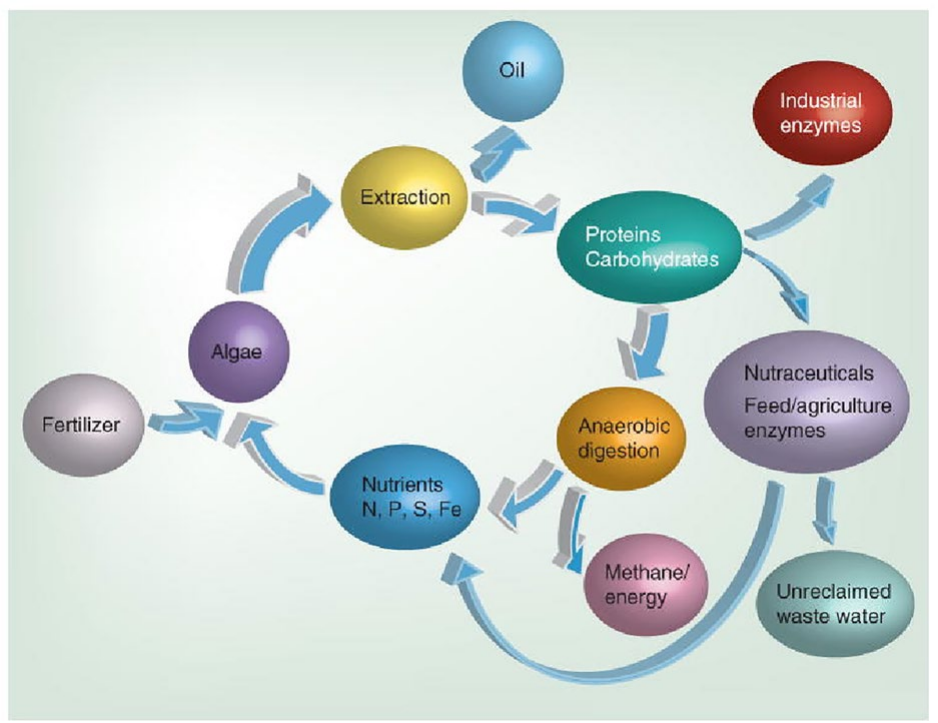
Carbon cycle.

In recent years, biodiesel has gained popularity as an environmentally friendly alternative to traditional fossil fuels. The main reactant for the production of biodiesel includes fatty acids such as residual cooking oil, tallow, animal fats, or lipids derived from microalgae. Also, nonedible oil source like soybean oil^5^, jatropha seed oil ^6^, yellow oleander seed oil^7^ are used to produce biodiesel. Also hybrid oil feedstocks ^8^ like quinary oil mixture (soybean oil, sunflower oil, canola oil, jatropha oil and pongamia oil) ^9^ are been explored in recent years to produce biodiesel using transesterification. Also, microalgae are minuscule photosynthetic microorganisms that possess a remarkable ability to produce a large amount of biomass and have a high concentration of lipids, which makes them a promising candidate for biodiesel production. In recent decades, several microalgal species have been examined because to their significant lipid content. Nutrient restriction has emerged as the most widely used approach to enhance microalgal lipid production and the carbon cycle due to the use of lipids from microalgae is shown in Fig. 1. The composition of the fatty acid esters that make up biodiesel has a notable influence on its characteristics. The fatty acid content of a particular strain of microalgae might vary significantly depending on the circumstances in which it is grown. The presence of saturated and monounsaturated fatty acids in biodiesel enhances its fuel characteristics. Both microalgae and macroalgae may be cultivated rapidly on a big scale. Microalgae have the potential to be utilised as energy crops because of their capacity to perform photosynthesis and their ability to get nutrients from organic matter. They have the ability to produce a multitude of valuable economic compounds, including as oils and fats. Following combustion, the environment is likely to remain uncontaminated as algal biofuel lacks any harmful constituents. Algae species contain a wide variety of oils. Some species have demonstrated elevated amounts of fatty acids. Some algae contain a greater proportion of fatty acid components in their dry mass^10^.

Transesterification is the primary method used to turn fatty acids into biodiesel. Fatty acids and alcohol react with a catalyst to form fatty acid esters and glycerine in this process. Biodiesel manufacturing has been on the rise in recent years as it climbed by about 30% between 2014 and 2022 (from 27.84 to 75.82 million tons each year). Transesterification, which involves heating a mixture of fatty acids and alcohol in the presence of a homogeneous catalyst, is the most widely used technique for producing biodiesel. As a result, efforts should be made to develop eco-friendly and effective heterogenous catalysts to overcome the issues in biodiesel manufacture, such as environmental contamination and equipment corrosion due to use of homogenous catalyst. Heterogeneous catalysts which are normally employed in biodiesel production includes post-harvest agro wate derived catalyst^11^, agricultural waste-based heterogeneous catalysts^12^, CaO derived from biomass^13^, catalyst derived from calcination of *Heteropanax fragrans* (*Kesseru*)^14^, zeolitic imidazole framework—8 Metal organic framework derived CaO/ZnO^15^, metal organic framework based catalyst^16^, sulfamic acid modified UiO-66 metal–organic framework^17^, *Musa champa* peduncle waste-derived catalyst^18^, catalyst from post harvested waste materials from the *Bharatmoni* banana plant^19^. These catalyst are regenerable, exhibit low corrosion rates and high catalytic activity^10^.

In the current study the catalytic activity of DESs synthesized were investigated for the transesterification of microalgae lipid into biodiesel. The catalyst was characterised using XRD, FESEM, and FT-IR to examine the nanoparticle composition and surface morphology. Optimization studies for finding the better yield of biodiesel was done along with kinetic models’ investigation for the reaction using kinetics data and mass transfer studies.

## Materials and methods

### Chemical and reagents

*Camellia sinensis* leaves purchased from Tea Dips, Chennai, Tamil Nadu. All the chemicals used are of AR quality, purchased from Uma Scientific suppliers, Kanchipuram.

### Synthesis of iron based nanoparticles

60 g of dried green tea leaves is dissolved in 1 L demineralised (DM) water and is boiled over a heating mantle. The green tea extract solution was vacuum filtered while the sludge was discarded. Ferrous sulphate solution of 0.1 N is prepared by dissolving 27 g in 1 L deionized water. Green tea extract and ferrous sulphate solution are mixed in a ratio of 1:2 which results into a black coloured precipitate solution. "Nanoparticles made of iron are produced by heating the solution to 700 °C until all the water has evaporated. When the precipitation mixture is heated the oxygen content present in the solution decreases which will thereby reduce the formation of iron oxide. The particles are then washed with ethanol and deionized water to avoid the oxidation of iron^20^. The prepared nanoparticles are characterised by SEM, FT-IR and XRD.

#### Extraction of algal oil

It took 5 days of sun drying in the sun for the azolla that has grown in water to become crisp while still retaining its green colour. The dried azolla is crushed into powder. The azolla is introduced to soxhlet apparatus. The solvent used for the extraction of algal oil from azolla is ethanol. The ethanol along with the algal oil was obtained from the process. The sample along with ethanol is fed into rotary evaporator and it is operated for 20 min, so that the oil is removed from the ethanol and stored separately^21^. The ethanol can be reused for future extraction purpose.

#### Transesterification process

The obtained algal oil is mixed with methanol and a catalyst in the ratio of 1:2. The apparatus used is round bottom flask, condenser and heating mantle. The solution containing methanol, algal oil and catalyst is poured in the flask and kept in mantle at a temperature of 60 °C and the condenser is fixed in top of flask and cooling water is supplied via hose pipe to the condenser. With the support of a magnetic hot plate stirrer, all of the designed tests were carried out at a maintained temperature of 60 °C. About 120 min of processing time is required. A separating column is used to let the produced solution settle for 20 h^22,23^. The biodiesel yield was assessed by gravimetric technique. The base layer containing Fatty acid methyl ester (FAME) was filled into a petridish, which was dried in an oven at 60 °C for 10 h. The remaining liquid was weighed as biodiesel mass. FAME content of the product was estimated using GC–MS Rtx®-5MS (30 m × 0.25 mm I.D. df = 0.25 μm, Restek Corporation). The fatty acids were recognized by relating the retention times of standard fatty acids and the composition was found using the corresponding peak areas in the chromatogram^24^.

After the ending of reaction, the nanocatalyst were separated by filtration. To consider the viability of reuse of the catalyst, its regeneration studies were examined based on the biodiesel yield at the optimal parameter conditions. At the end of each cycle, the catalyst was washed with ethanol and dried at 100 °C for 4 h.

## Results and discussions

### Characterisation

**Figure 2:**
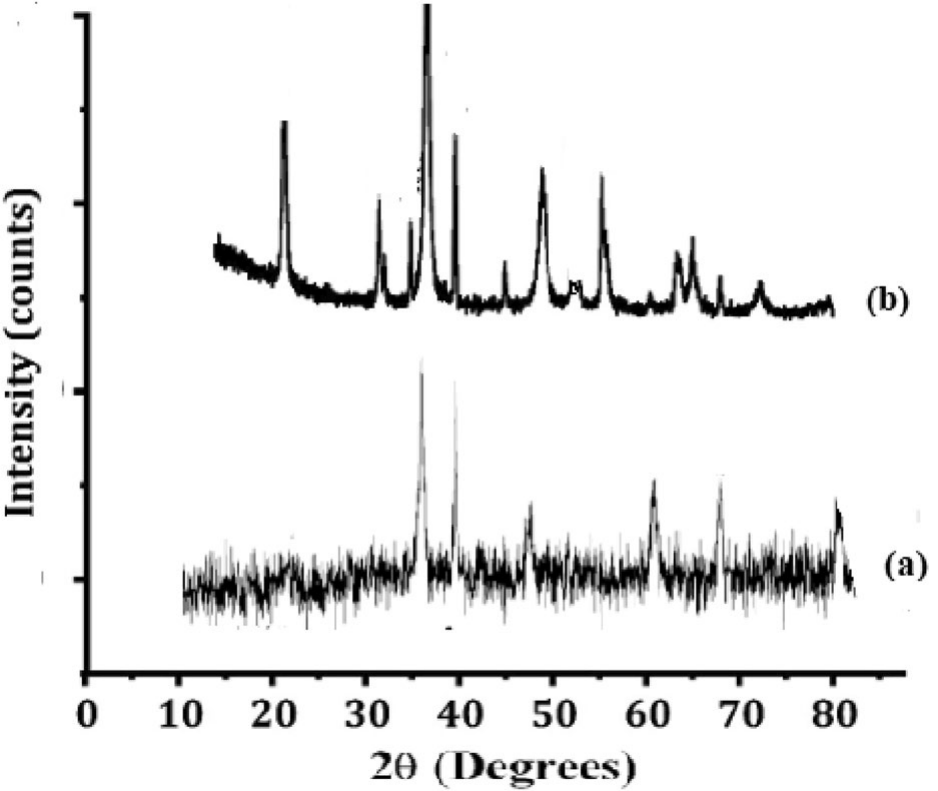
XRD indication of iron NPS. (**a**) Before calcination (**b**) After calcination.

Figure 2 depicts the formation of these oxides and hydroxyls occurred as a result of the oxidation of the iron nanoparticles during their synthesis^25^. After calcinations shows weak reflections at 2θ values of 35.30° and 62.55° which is attributed to either Fe_3_O_4_, or Fe_2_O_3_ (JCPDS file no. 85-1436/25-1402/85-0987). The XRD exhibited peaks correspond to the (012), (110), (113), (024), (018), (300). The nanoparticle size is estimated using Debye-Sherrer formula.

$${\text{D}} = { }\frac{{0.94{\uplambda }}}{{{\text{Bcos }\theta}}}.$$

where, 0.94—shape factor, λ—x-ray wavelength, B—line broadening at half the maximum intensity in radians, and θ—Bragg angle. The mean size of nanoparticles was found to be 20 nm. The size of the particles ranges from 30 to 120 nm. However, the particles beyond 100 nm are very less. The average percentage of particles in the synthesis is 75 nm^26^.

**Figure 3:**
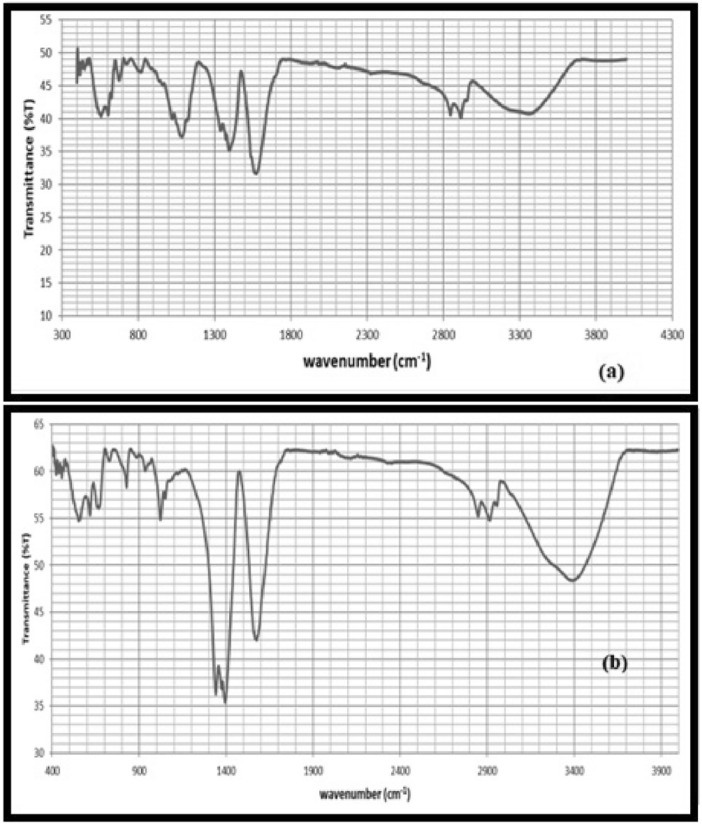
FTIR spectra for iron NPs. (**a**) Before calcination. (**b**) After calcination.

The FTIR spectra of the NPs are shown in Fig. 3. The band positions near 3400 cm^−1^ and 1630 cm^−1^ attributed to O–H stretching, due to water molecules. The as prepared iron oxide shows a band at 3403 cm^−1^ due to N–H stretching. The band at 1715 cm^−1^ is attributed to carbonyl stretching. The band at 1469 cm^−1^ corresponds to C–N stretching. The peak at 1015 cm^−1^ are attributed to the bending vibrations of N–H groups. The sample also shows bands at about 590 cm^−1^ and 430 cm^−1^ attributed Fe_2_O_3_.

Figure 3b depicts the bands at about 540 cm^−1^ and 460 cm^−1^ in the calcined iron oxide samples. The bands at 3398 cm^−1^, 1636 cm^−1^, and 1039 cm^−1^ correspond to O–H, C=C, and C–O–C stretching vibrations, in that order. This suggests that the iron NPs were reduced and stabilised by the green tea polyphenols and caffeine^25,27^. The 460 and 546 cm^−1^ adsorption bands were found to match to the Fe–O lengths of Fe_2_O_3_ and Fe=O_4_, respectively.

**Figure 4:**
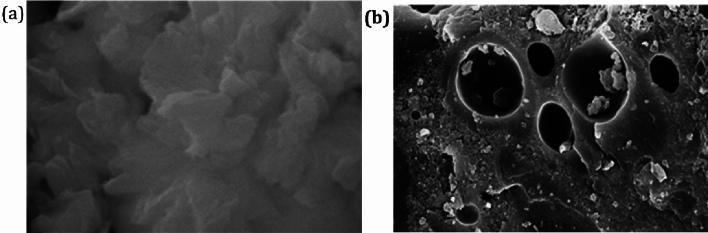
FESEM image of the iron nanoparticles. (**a**) Before calcination. (**b**) After calcination.

The analysis was done for studying the surface morphology of the adsorbent. FT-IR equipment. The FESEM image of the iron nanoparticles before and after calcination is shown in the Fig. 4. Before calcination the FESEM image shows the surface appearing relatively rough or uneven (Fig. 4a). After calcination a large number of pores were seen in the FESEM images of the iron nanoparticles The size of the iron nanoparticles can be varying which can be attributed to the oxidation of them when exposed to atmosphere but however they do not agglomerate because iron nanoparticles act as capping and stabilizing agent thereby protecting the zerovalent iron and iron oxides (Fig. 4b)^28^.

**Figure 5:**
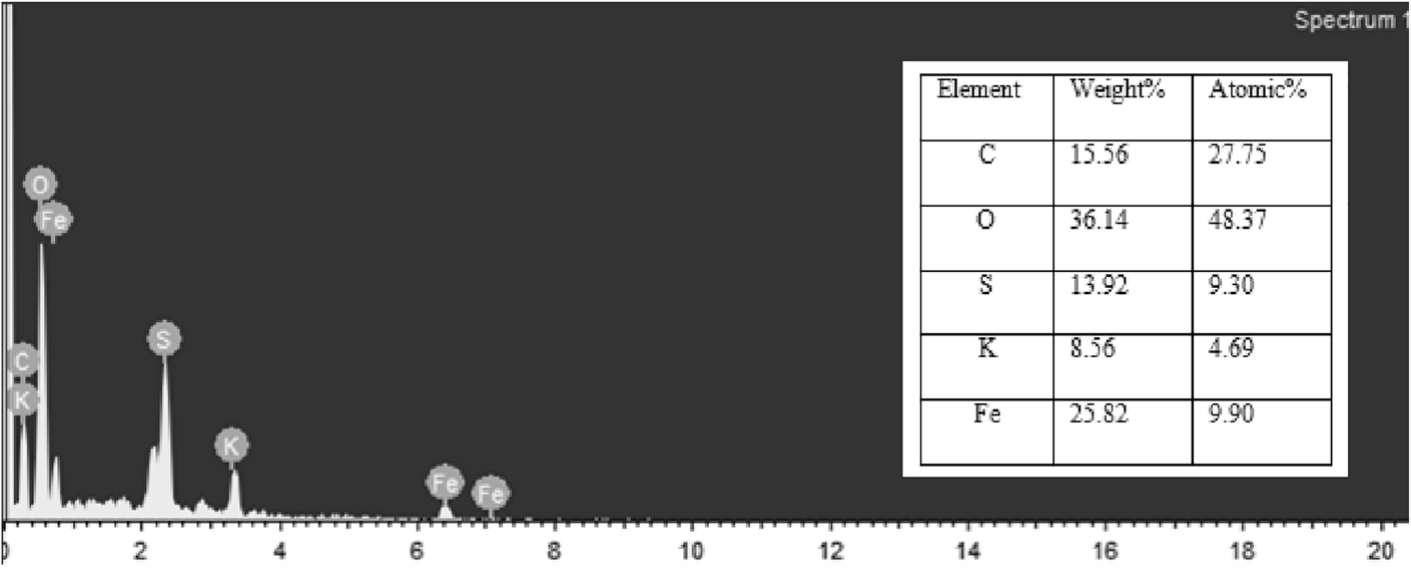
EDX of catalyst.

Figure 5 shows the composition of the components in the elemental form. The components of the adsorbent are C, O, S, K, Fe with composition of 15.56%, 36.14%, 13.928%, 8.564%, 25.82% respectively. Since ferrous sulphate is used to create iron nanoparticles, there is sulphur present. The polyphenol component of the green tea extract contributes to the high composition of oxygen content^1^.

**Figure 6:**
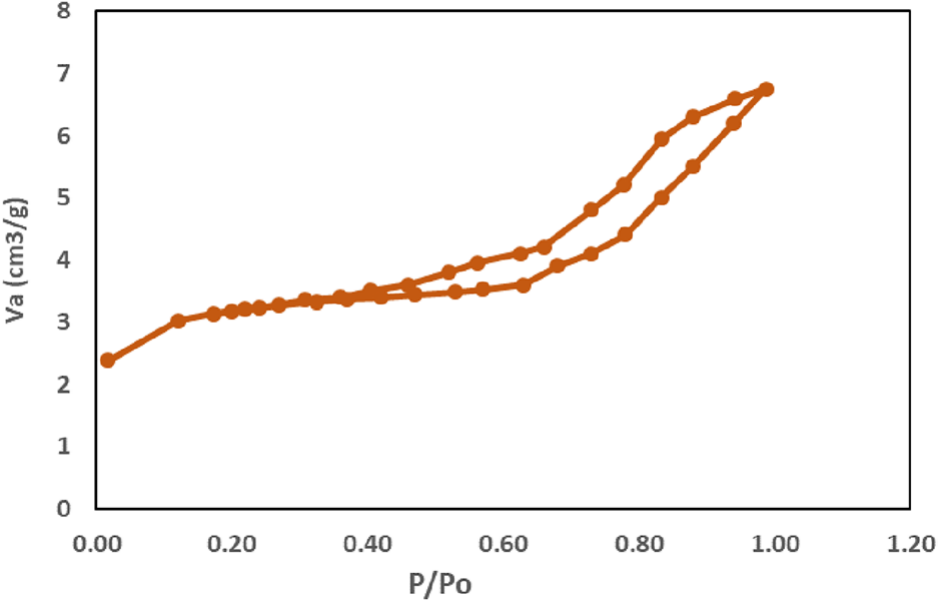
BET isotherm of catalyst.

As observed in the EDAX spectrum, the adsorption surface area and pores size were measured using a BET (Brunauer-Emmet-Teller) surface area analyzer prior to the adsorption–desorption investigation of N_2_ (Fig. 6). Plotting nitrogen multilayer adsorption versus relative pressure allows one to use adsorption/desorption processes to measure pore area and specific pore volume^29^. The BET investigation shows that the catalyst has a surface area of 7.84 m^2^ g^−1^, with a mean pore diameter of 8.95 nm and a pore volume of 0.12 cm^3^ g^−1^.

BET (Brunauer-Emmet-Teller) surface analyzer was used for the N_2_ adsorption–desorption investigation, as the EDAX spectrum prior to the adsorption surface coverage and pores size investigations. The adsorptive/desorptive processes of nitrogen multilayer adsorption may be utilised to determine the specific pore volume and pore area as an equivalent of relative pressure. Based on BET analysis, the catalyst has a surface area of 7.84 m^2^ g^−1^, a pore volume of 0.12 cm^3^ g^−1^, and an average pore diameter of 8.95 nm. Glycerine and ester are the final liberations of trans-esterification process. To separate ester as well glycerine, phased separation can be used. After the mix has set, the last trace of glycerine is recovered by adding wash water at a volumetric rate of 5%. The washing cycle is repeated until the ester layer is clear^30,31^.

### Extraction of algal oil

In the mechanical extraction most of the oil was present in the residual cake so that the solvent extraction is preferred since the quantity of oil content was more. Extraction is repeated numerous times by refluxing the ethanol using a siphon and condenser^32^. The oil yield was determined using the equation given by

$$Oil\,Yield\,(in\,\% ) = \frac{{W_{oe} }}{{W_{cs} }}X100$$

where W_oe_ corresponds to oil content expelled (g), and W_cs_—dry azolla content (g). The properties of extracted oil, is presented in earlier literature^22^ and some of them are presented in Table 1.

**Table 1:** Property estimation of extracted oil.

Parameter	Property
Free fatty matter (wt %)	1.75%
Flash point	109 °C
Acid value	1.37 mg KOH/g oil
Dynamic viscosity at 40 °C (mm^2^ s^−1^)	46

### Molar ratio impact on yield

One among most notable parameters determining the yield as well productive cost for the conversion of oil to biodiesel is molar ratio. Since the process is reversible and the oil-to-alcohol stoichiometric ratio is 1:3, higher molar ratios are required to improve miscibility and interaction between the alcohol and the triglyceride. To convert triglycerides into biodiesel, it is mandatory to disseminate the bonds between glycerine as well fatty acids, which requires an excess of methanol. The influence of molar ratio over yield was analysed for several ratios 1:6, 1:9, 1:12, 1:15. By varying one variable at the time and kept other parameters constant such as reactive temperature at 60 °C, reaction duration at 120 min and holding 1% optimum value of catalytic amount with mass of the oil along with percentage variation in yield has been experimented.

**Figure 7:**
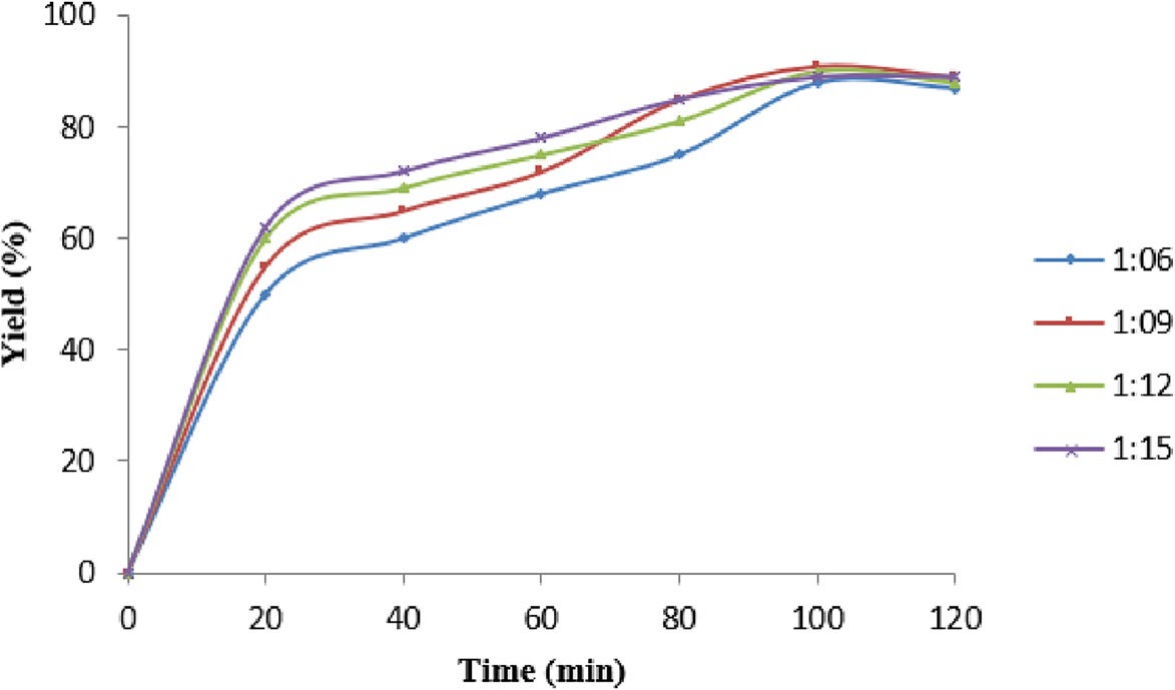
Effect of molar ratio of oil to methanol in biodiesel yield Experimental conditions: the reaction was run at a oil to methanol ratio of 1:6 to 1:15, 1% of catalyst loading, reaction time 120-min, and reaction temperature of 60 °C.

Alkyl ester conversion is improved with higher molar ratios. In addition, there are three distinct phases to the processes as a whole: mass transfer, kinetics, and equilibrium control. The mass transfer step moves at a snail's pace due to the fact that triglycerides and methanol are incompatible^33^. Charging an excess of methanol might boost biodiesel generation by shifting the balance to the forward side. The effect of this ratio on yield is seen by Fig. 7, where the rate reaction rate increased as the molar ratio of oil to methanol was increased. There was an increase in biodiesel output from a 1:6 to a 1:15 molar ratio. This led to the conclusion that a molar ratio of 1:9 was optimal; above this, glycerol separation became more problematic due to the residual glycerol in the biodiesel phase. Furthermore, the alcohol requirement for inedible oils is higher than for edible oils in order to achieve maximum ester yield. However, in inedible oil, glycerol output was greater and ester content yield was lower^34,35^.

### Effect of reaction temperature on yield

**Figure 8:**
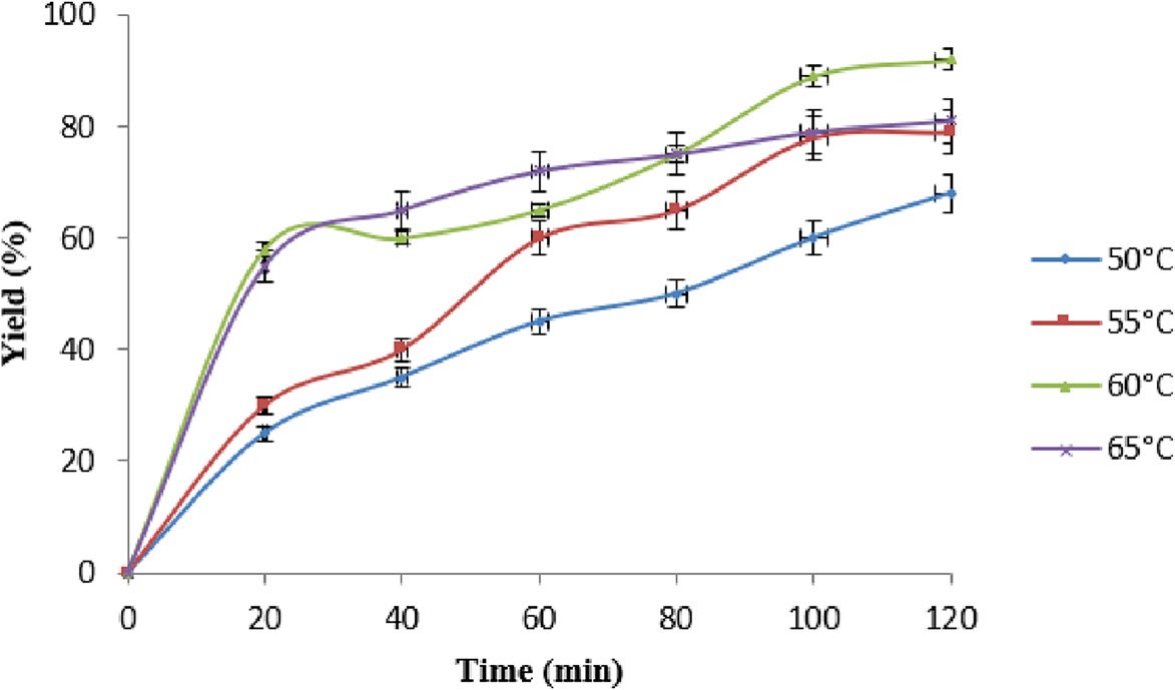
Effect of reaction temperature in biodiesel yield Experimental conditions: the reaction was run at a oil to methanol ratio of 1:9, reaction time 120-min, 1% of catalyst loading and reaction temperature of 50–65 °C.

Activation energy, reaction rate, as well yield are significantly influenced by the temperature of the process. Holding molar ratio and catalytic load to be constant on their optimum value of 1:9 and 1% by weight and variation of reactive temperature from 50 to 65 ℃ were used to assess the impact of temperature on yield^36^ (Fig. 8). The rate was meagre at lower temperatures, and the output was just 30–65 percent after 40th min of such reaction envisaged at 50 °C. Rising reaction temperatures boost yields up to 65 °C, beyond which further increases in temperature have little effect on yield.

This could be due to methanol loss at high temperatures, which inhibits the reaction at the three-phase interface. Higher temperatures cause rapid molecular movement, which increases the kinetic energy of reacting molecules and thus increases collisions between reacting molecules^28^. The heat produced by the collision of these reacting molecules is used to convert oil into biodiesel. Raising the temperature accelerates the process due to an increase in kinetic energy. Increased temperature mostly benefits a quicker response time. It was found that a reaction temperature between 60 and 65 °C produced the highest yield.

### Effect of catalyst: oil ratio on yield

**Figure 9:**
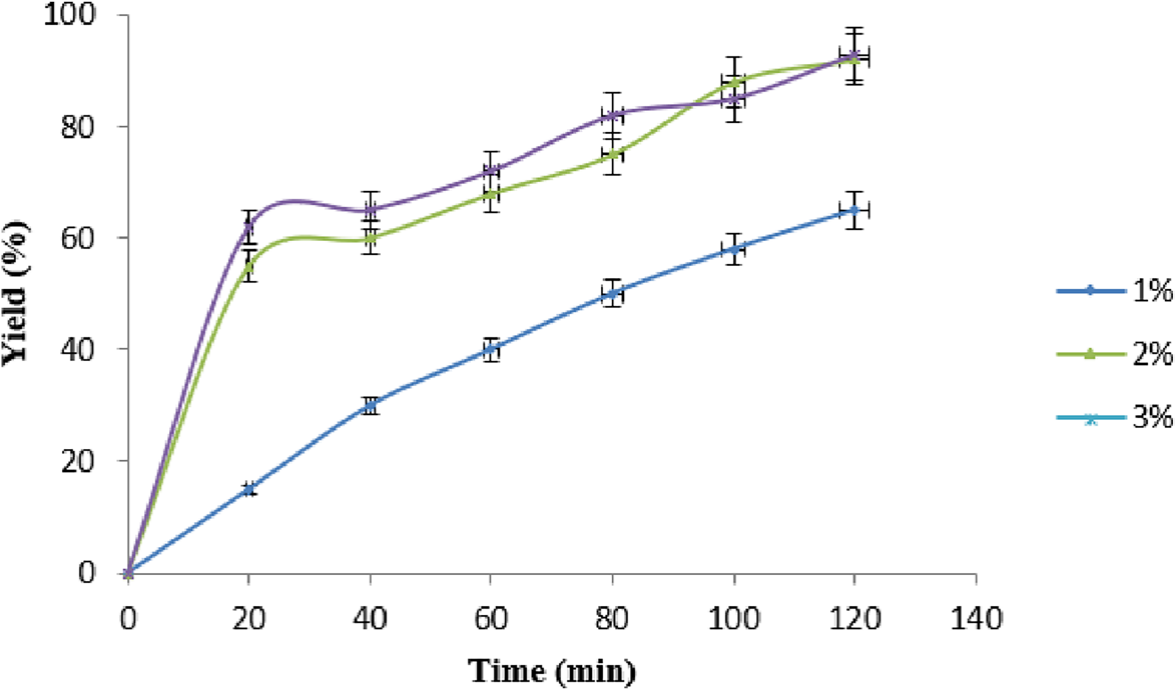
Effect of catalyst loading in biodiesel yield Experimental conditions: the reaction was run at molar ratio of 1:9, reaction time 120-min, and reaction temperature of 60 °C with varying catalyst load.

A large surface area and strong basic sites should make catalysts very active. We looked at what happened when we went from a 1 to 3% GO nanocatalyst to azolla oil weight ratio increase and keeping the other parameters constant such as the reactive heating level at 60 °C, reaction duration at 120 min and oil—methanol ratio at 1:9^37^. The addition of GO nanocatalyst increased the yield of biodiesel. The addition of 2% nanocatalyst resulted in the highest yield. There is no discernible difference in yield between 2.5 and 3% catalyst weight ratio. The results show that the catalyst's addition increased the reaction's active area, resulting in a faster response rate and a larger yield (Fig. 9). The use of excessive catalyst will cause the formation of slurry so that inhibit the formation of biodiesel thereby it attained the saturation^27,38^.

### Kinetics studies

#### Langmuir Hinshelwood-Hougen-Watson model

On their publication, Yang et al.^38^ established a generic rate equation that is grounded in the LHHW mechanism. This rate equation is derived with the presumption that the alcohol and adsorbed triglyceride surface reaction is the step that determines the rate^39^. We may express this rate equation as follows:

$${\text{Step - 1}} \;\; {\text{Triglycerides }} + {\text{CH}}_{{3}} {\text{OH}} \rightleftharpoons {\text{Diglyceride }} + {\text{ Methyl ester}}$$

$${\text{Step - 2}} \;\; {\text{Diglyceride }} + {\text{ CH}}_{{3}} {\text{OH}} \rightleftharpoons {\text{Monoglyceride}} + {\text{Methyl ester}}$$

$${\text{Step - 3}}\;\; {\text{Monoglyceride }} + {\text{ CH}}_{{3}} {\text{OH}} \rightleftharpoons {\text{Glycerine }} + {\text{ Methyl ester}}$$

$$r = \frac{{k_{f} k_{T} k_{A} \left[ T \right]\left[ A \right] - k_{r} k_{D} k_{e} \left[ D \right]\left[ E \right]}}{{\{ \left( {1 + k_{T} \left[ T \right] + k_{A} \left[ A \right] + k_{D} \left[ D \right] + K_{E} \left[ E \right] + K_{M} \left[ M \right] + k_{G} \left[ G \right]} \right)\}^{2} }}.$$

where are kf and kr, forward and reverse reaction rate constant of the rate determining step. kT, kA, kD, kE, kM, kG are the equilibrium constants for adsorption of triglyceride, alcohol, diglyceride, fatty acid, methyl ester, monoglyceride ang glycerine respectively. At very short initial times of reaction, assume [D]≈ [E] ≈ [M] ≈ [G] ≈ 0.

**Figure 10:**
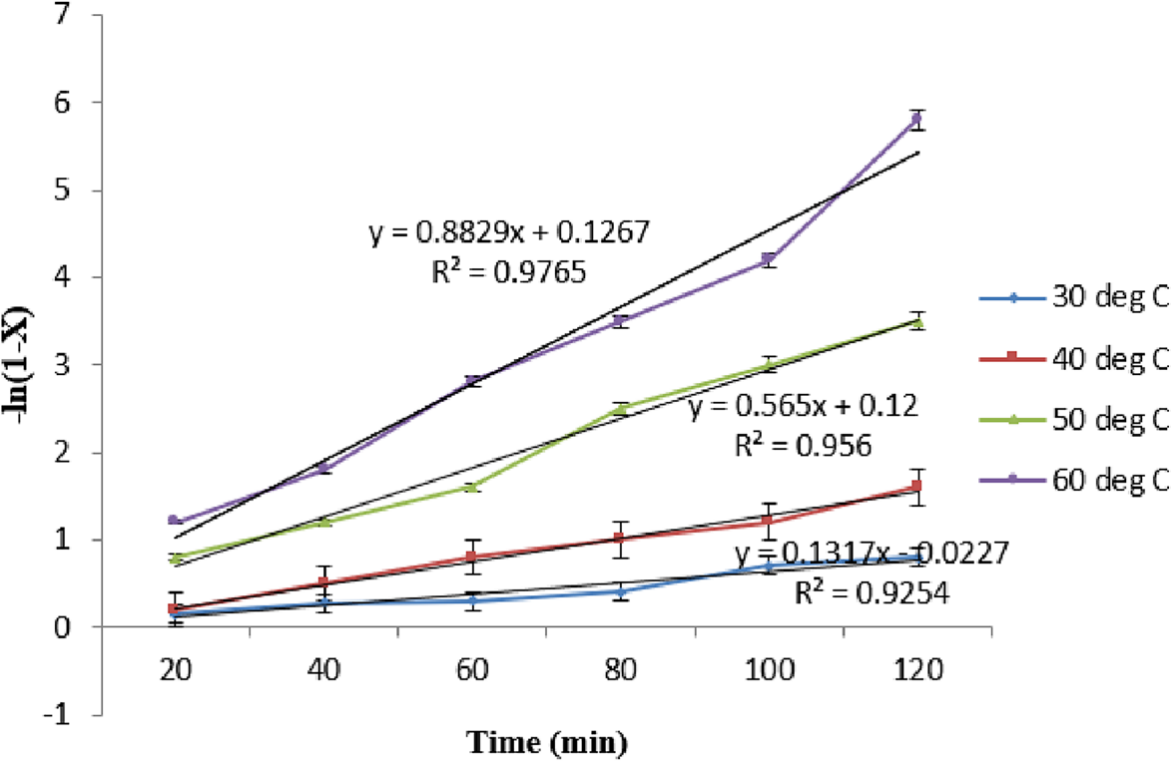
Plot of −ln (1−x) vs time at different temperature [15:1 methanol to oil molar ratio, 5 wt.% catalyst].

The Pseudo First Order kinetics model is used to the aforementioned equation in the case of very high beginning alcohol concentrations^40^. The kinetics of catalysed methanolysis were studied at various temperatures; the corresponding graphs between—ln(1−X) vs. t are shown in Fig. 10. The fact that transesterification uses a significant quantity of alcohol than the required stoichiometric molar ratio—leads many people to conclude that the reaction is pseudo first order. Temperatures ranging from 30 to 60 °C were used to conduct reactions in order to determine the activation energy^41^.

$$\begin{aligned} {\text{Rate}} & = \frac{ - d\left[ T \right]}{{dt}} \\ - \ln (1 - {\text{X}}) & = {\text{kt}} \\ {\text{k}} & {\text{ = Ae}}^{{ - {\text{Ea/RT}}}} \\ \end{aligned}$$

**Figure 11:**
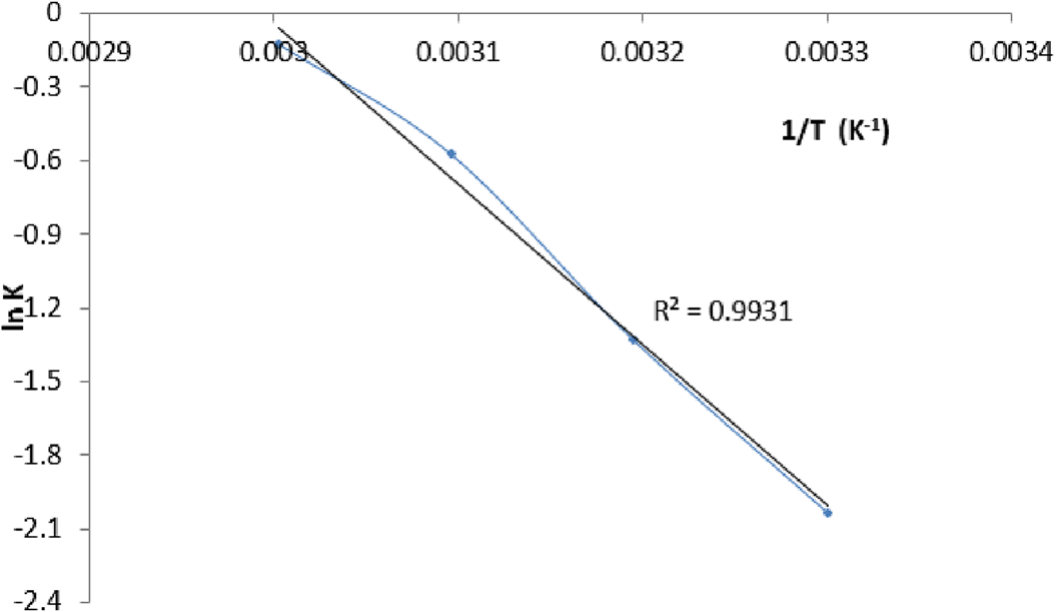
Arrhenius plot of transestrification.

Here, k stands for the rate constant (min^−1^), X for the oil to FAME conversion at time t, Ea for the activation energy (kJ mol^−1^), A for the pre-exponential factor (min1), R for the gas constant (8.314 * 10^−1^ kJK^-1^ mol^−1^), and T for the reaction temperature (°K). It was clear from these plots that the methanolysis rate constants at 60 °C were 0.762 min^−1^. The activation energy (Ea) and pre-exponential factor (A) were calculated using the Arrhenius model. A plot of lnk vs 1/T in Fig. 11 was used to calculate the values of Ea and A for methanolysis, which were found to be 52.5 kJ mol^−1^ and 325.2 × 106 min^−1^, respectively^42^. The activation energy for methanolysis in the presence of heterogeneous catalysts that has been reported in earlier research is compared to the current finding. Based on the comparison, it was found that the methanolysis activation energy was detected to be within the range of values reported for heterogeneous catalysts. This comparison indicates that the reaction rate during transesterification is much greater for base catalysts^43^.

### Eley Rideal mechanism

Multilevel reaction-based kinetics was the model chosen for this investigation. The irreversible reaction was modelled through six kinetic constant rates^44^.

$$\begin{gathered} {\text{Step - 1}}\;\;{\text{Triglycerides }} + {\text{CH}}_{{3}} {\text{OH}} \underset{{{\text{k2}}}}{\overset{{{\text{k1}}}}{\rightleftharpoons}} {\text{Diglyceride }} + {\text{ Methyl ester}} \hfill \\ {\text{Step - 2}}\;\; {\text{Diglyceride }} + {\text{ CH}}_{{3}} {\text{OH}} \underset{{{\text{k4}}}}{\overset{{{\text{k3}}}}{\rightleftharpoons}} {\text{Monoglyceride}} + {\text{Methyl ester}} \hfill \\ {\text{Step - 3}}\;\; {\text{Monoglyceride }} + {\text{ CH}}_{{3}} {\text{OH}} \underset{{{\text{k6}}}}{\overset{{{\text{k5}}}}{\rightleftharpoons}} {\text{Glycerine }} + {\text{ Methyl ester}} \hfill \\ \end{gathered}$$

$$\begin{gathered} \frac{d[T]}{{dt}} = - k_{1} [T] + k_{2} [E] \hfill \\ \frac{d[D]}{{dt}} = k_{1} [T] - k_{2} [E] - k_{3} [D] + k_{4} [E] \hfill \\ \frac{d[M]}{{dt}} = - k_{3} [D] - k_{4} [E] - k_{5} [M] + k_{6} [E] \hfill \\ \frac{d[E]}{{dt}} = k_{1} [T] - k_{2} [E] + k_{3} [D] - k_{4} [E] + k_{5} [M] - k_{6} [E] \hfill \\ \end{gathered}$$

**Figure 12:**
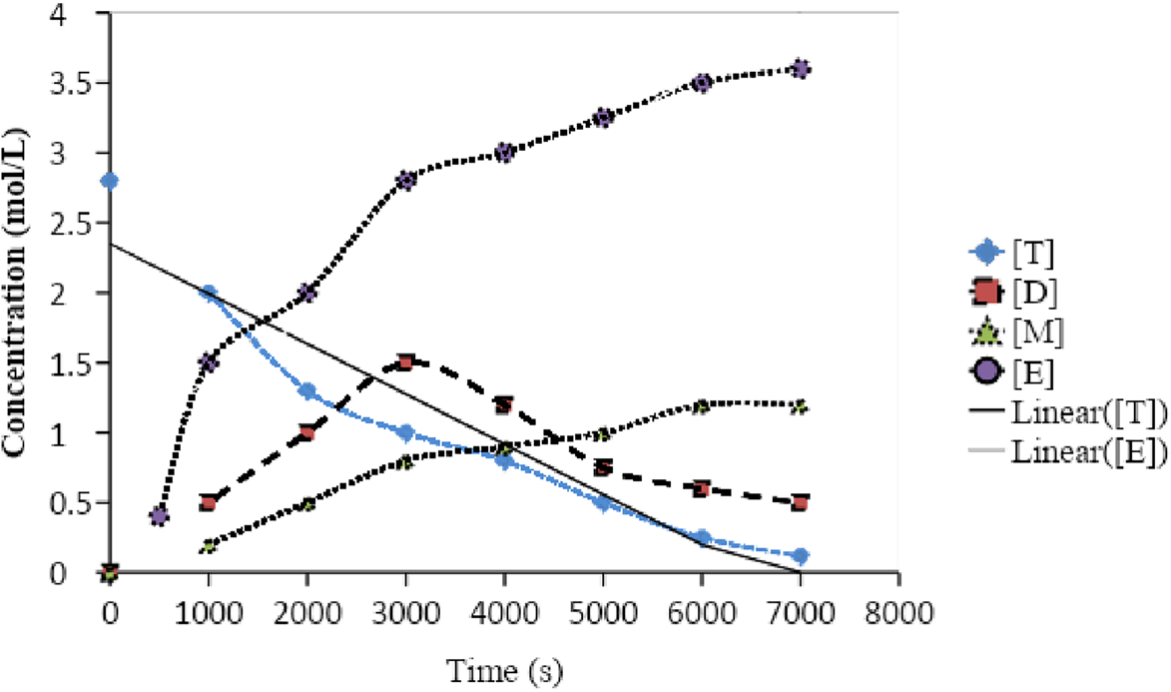
Eley–Rideal reaction scheme.

The kinetics parameter was estimated using the curve fitting method. Six kinetic constants must be calculated using the Eley–Rideal reaction scheme (Fig. 12). By comparing the experimental data with the model equations, we may numerically estimate the kinetic rate constants^45^. Table 2 shows the kinetics parameter constant obtained for the transesterification reaction based on curve fittings. The value of the kinetic parameter constant was calculated in simulation using the Eley–Rideal method^46^.

**Table 2:** Kinetics parameter constant for transesterification reaction based on curve fittings.

Constants (s^−1^)	k_1_	k_2_	k_3_	k_4_	k_5_	k_6_
Value	7.87 × 10^–3^	4.87 × 10^–3^	6.65 × 10^–3^	1.26 × 10^–4^	3.21 × 10^–4^	1.2 × 10^–4^

### Catalyst performance evaluation

The potentiality of the catalysts was determined using their turnover frequency (TOF). During TOF calculation, the total alkalinity of the catalysts was considered, and the molecular weight (MW) of the FAME was assumed to be one-third that of the algal oil.

$${\text{Yield }}\left( {{\% }} \right) = { }\frac{{\text{weight of biodiesel produced}}}{{\text{ weight of oil used}}} \times { }100$$

$${\text{TOF}} = { }\frac{{\text{moles of FAME produced (mmol)}}}{{\text{ catalyst alkalinity (mmol) x time (h)}}} \times { }100$$

The TOFs of catalysts were found to be 18.31 h^–1^ . These results reveal that the highest TOF, indicating its highest efficiency during the transesterification reaction. A similar observation was reported^47^ for Al_2_O_3_ catalysts with TOFs of 43.2 h^–1^. Conversely, the lowest TOF reported of 6.59 h^−1^ in their biodiesel synthesis study, for catalyst derived from sugarcane bagasse ash^6,55^. Furthermore, for*Heteropanax fragrans* ash solid catalyst, an even lower TOF of 0.59 h^−1^ was observed^14^.

### Biodiesel characterization

**Figure 13:**
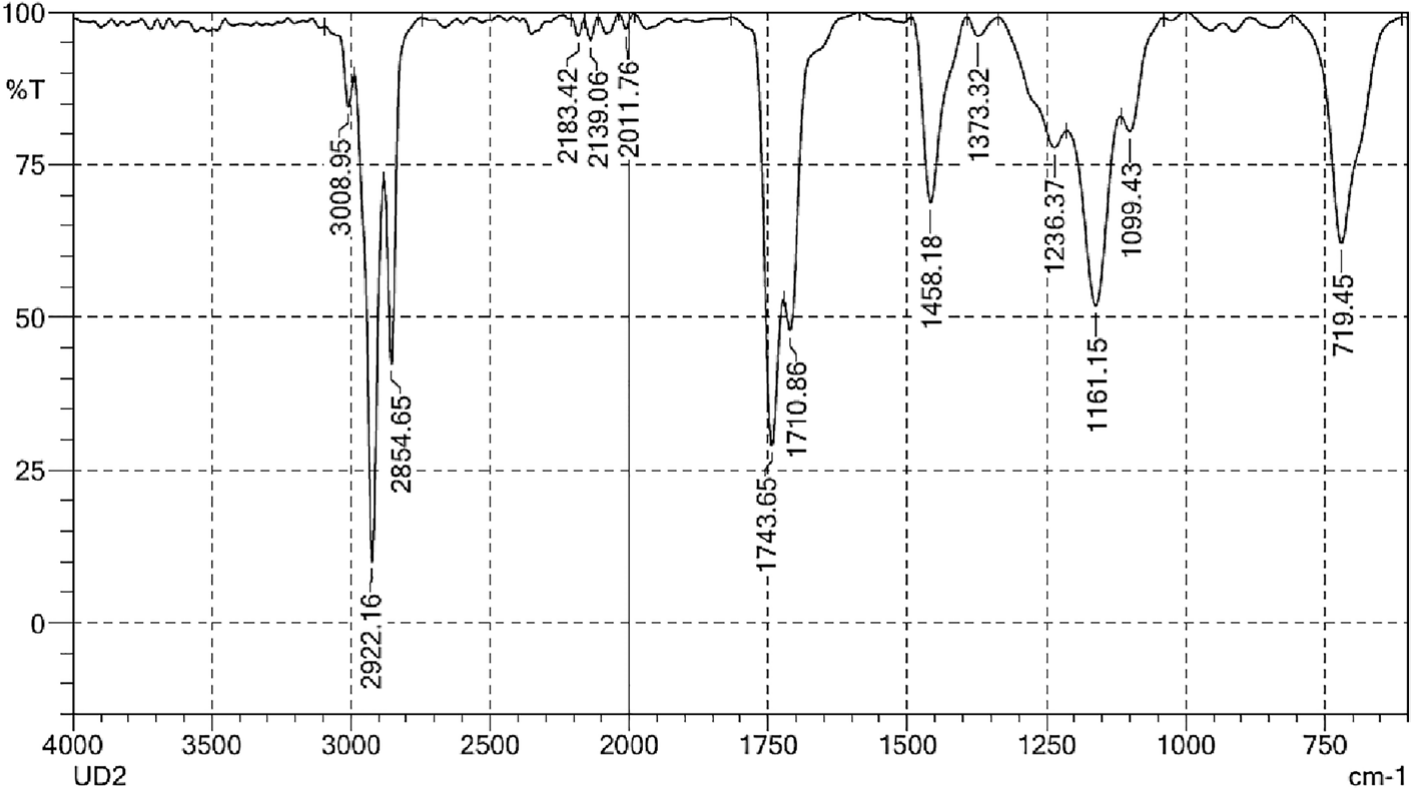
FT-IR spectra of biodiesel.

The biodiesel FT-IR spectrum (Fig. 13) shows peaks between 3600 and 400 cm^−1^. The absorbance band is seen in the biodiesel spectrum. Figure 3 shows the FTIR Spectra of azolla biodiesel, which show regions of strong absorption between 800 and 500 cm^−1^, 1800 and 1000 cm^−1^, and 3500 and 2500 cm^−1^. The CH_2_ stretching vibration of alkane complex is held notable for peak intensity seen at 2922 and 2854 cm^−1^. Fatty acid methyl ester is detectable at 1161 and 1099 cm^−1^. The methyl ester molecule’s C=O stretching vibration was detected at 1743 cm^−1^, whereas the -CH_2_ peaks were located at 719 cm^−1^.

**Figure 14:**
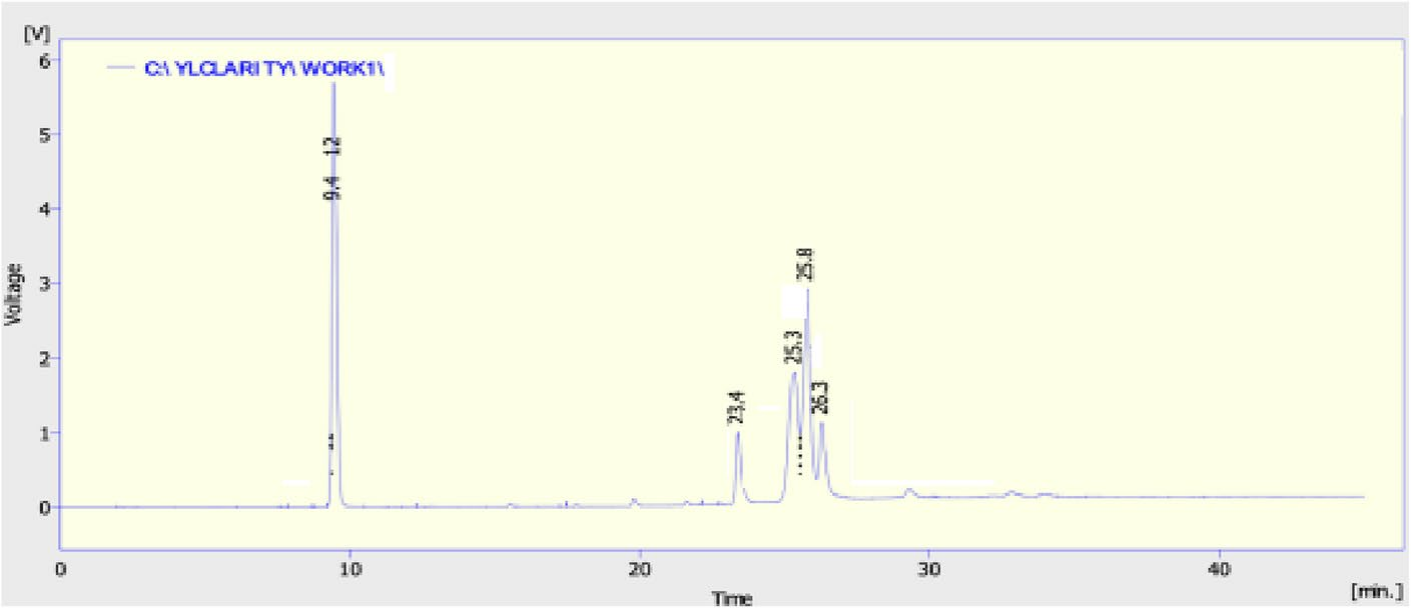
GC-FID of biodiesel.

Gas chromatography with flow ion detection was used to evaluate (Fig. 14) the chemical composition of *azolla* biodiesel (GC-FID). The composition of FAME (Fatty Acid Methyl Ester) is revealed by GC analysis, which confirms the presence of seven saturated and unsaturated acids. Saturated fatty acids that comprise methyl ester include butyric acid, myristic acid, palmitic acid, stearic acid, and magaric acid^48^. These acids are known for their ignite characteristics and strong oxidative stability. Because of their existence of double bond atoms, esters (including oleic acid, linoleic acid, and docosahexaenoic acid) are unstable in an oxidative environment and have a low energy content. Cloud and pour point, two cold properties of biodiesel, are affected by these unsaturated fatty acids^49^. The properties of biodiesel produced is given in Table 3.

**Table 3:** Biodiesel properties.

S.No.	Properties	Test method	Biodiesel standards ASTM D6751	Azolla filiculodies biodiesel value
1	Flash point (°C)	ASTMD93	93 to 170	145
2	Fire point (°C)	ASTMD93	106 to 180	159
3	Cloud point (°C)	ASTMD2500	− 3 to 15	12
4	Pour point (°C)	ASTMD97	− 5 to 10	9
5	Viscosity (cSt)	ASTMD6751	1.9 to 6	3.5
6	Calorific value (MJ kg^−1^)	EN14213	Min 35	32.25
7	Density (kg m^−3^)	ASTMD792	880	850

### Catalyst reusability studies

**Figure 15:**
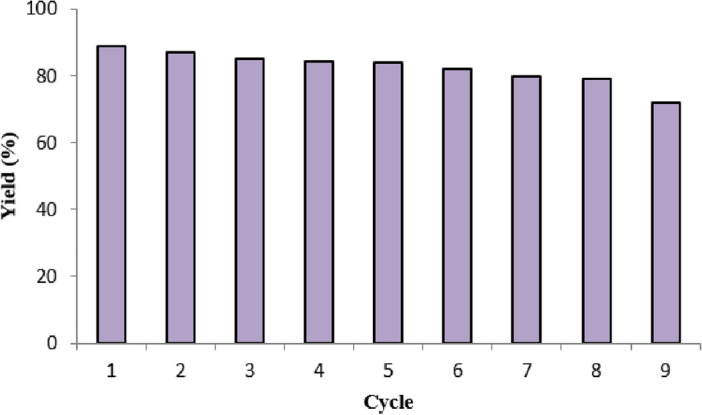
Regeneration and reusability of catalyst during biodiesel production.

Recovered solid catalyst was used in subsequent investigations conducted under identical operating conditions once batch studies were finished^53^. The biodiesel output was not noticeably diminished, and the GO catalyst continued to exhibit catalytic activity even after 6 cycles, as seen in Fig. 15. The loss of catalytic activity in the repeated studies might explain the modest decline in yield. The price of the catalyst is a major factor in the biodiesel pricing, from a financial perspective. As a result, stability and consistent activity are more important in industrial applications^54^. Table 4 summarises the results of the comparative study and some of the most recent research on nanocatalyst.

**Table 4:** Efficacy of several nanocatalytic components on biodiesel output.

Catalyst	Feed component	Yield (%)	References
Iron nanoparticles from *Camellia Sinensis*Leaves	*Azolla filiculodies* oil	91	This study
GO nanocatalyst	Phyoto-remediated *Azolla* oil	85	^22^
Perovskite (BaCeO_3_)	*Azolla pinnata* oil	98.4	^28^
Homogenous KOH	*Azolla Microphylla* oil	99	^34^
Homogenous KOH	*Azolla filiculoides*	54	^34^
Cr/Ca/γAl_2_O_3_	Cooking oil	92.7	^23^
KOH/ Fe_3_O_4_ @Al_2_O_3_	Canola oil	98.8	^48^
CaO/CuFe_2_O_4_	Chicken fat	94.5	^50^
zeolitic	*Spirogyra elongata*	72.7	^46^
ZnO catalyst from banana	Fish waste lipid	90	^51^
Zeolite supported ZrO_2_	Rubber seed oil	86.22	^44^
Alkaline modified zirconia catalyst	Waste cooking oil	79.7	^23^
Sodium hydroxide	Ulva lactuca Seaweed	87.8	^37^
Sodium hydroxide	*Azolla filiculoides* lipids	82.8	^52^
Chicken eggshell waste	Green Algae *Spirogyra*	96.18	^38^

**Figure 16:**
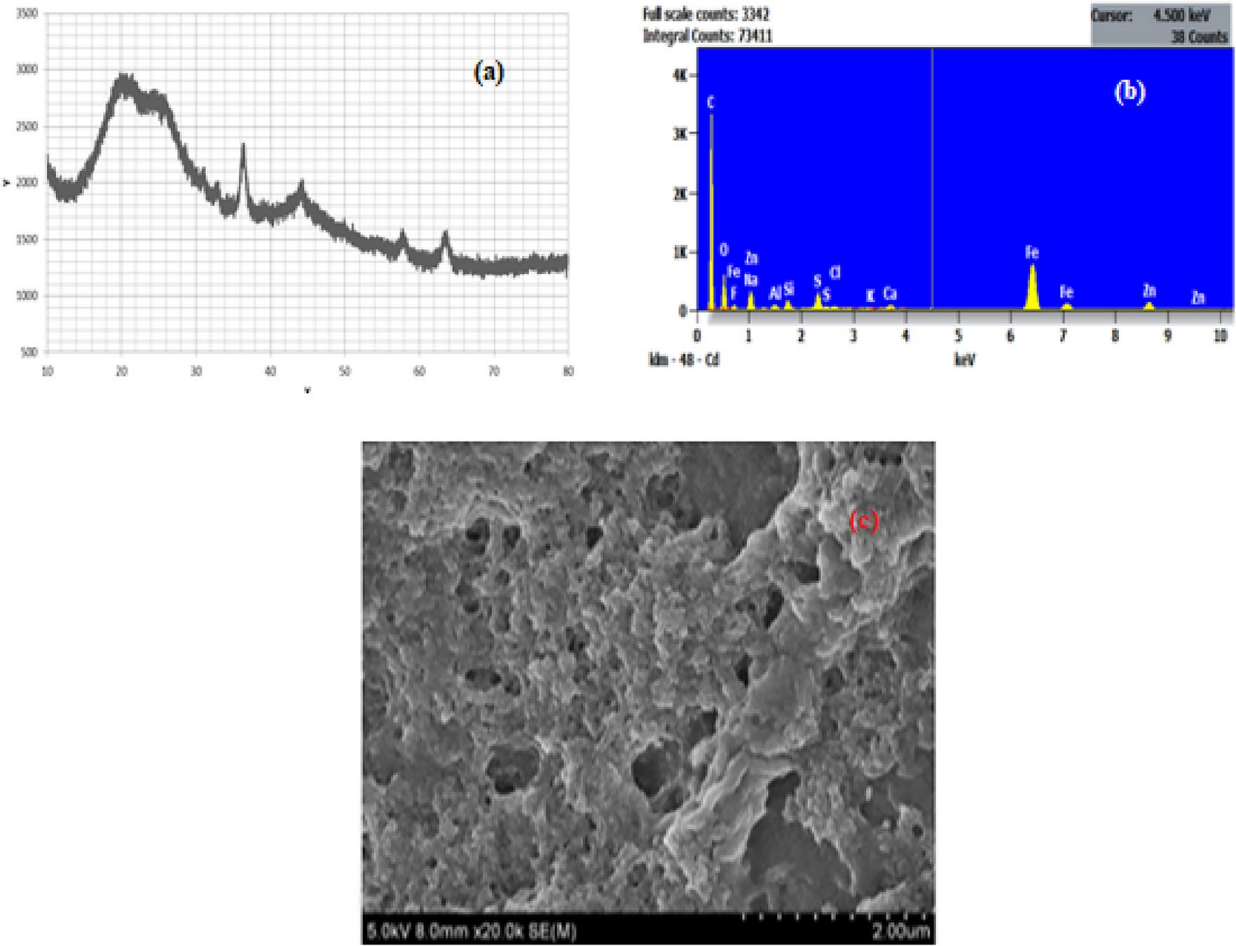
Regenerated catalyst characteristics (**a**) XRD, (**b**) EDS, (**c**) SEM.

Figure 16a depicts the XRD patterning proved the crystalline state of reused catalyst after 9th cycle. The peaks appearing at 2θ range of 20°, 35.70°, 49.45°, 57.55° and 63.90° are indexed to hkl (0 1 2), (1 1 0), (0 2 4), (0 1 8), (3 0 0) respectively. Such observations can be afforded to structures representing Fe-nanoparticles. The advent of greatest peak at 35.7° recognizes the occlusion of α-Fe_2_O_3_ (JCPDS: 033-0664).

Figure 16b is the EDS of the recycled nanocatalyst which shows that the particle consists of Fe/C/O with evaluated molar ratio 3/1/2. The elemental composition confirmed the formation of Fe^3+^ throught the line orientation of oxygen (0–2 keV). The appearance of peaks in the spectra betwixt the line orientation of 6 and 8 keV depicts the occlusion of elemental iron. The elemental traces were present in spectra possibly during the regeneration and along the process of biodiesel synthesis originated as impurities.

Figure 16c revealed the morphology of the recycled nanocatalyst representing clusters of roughed surface acclimations laced with numerous pores as well irregular shapes. The pores are visible with dimension varying from 20 to 30 nm which confirms that the recycled catalysts were nanocatalysts in nature.

## Conclusion

A nanocatalyst with an average pore diameter of 8.95 nm, a specific volume of 0.12 cm^3^ g^−1^, and a specific surface area of 7.84 m^2^ g^−1^ was shown to have exceptional catalytic activity, stability, and longevity in experimental research. The transesterification yield of azolla oil was more than 92% under optimal conditions, which included a 1:9 oil-to-methanol molar ratio, 2.5 weight percent nano-catalyst, a reaction temperature of 60–65 ℃, and a reaction length of 120 min. Compared to homogeneous catalysts, the synthesised catalyst had a greater catalytic activity. The results of the research suggest that biodiesel may be produced from macroalgae. Furthermore, the findings imply that algae, which is widely accessible and probably affordable, might be a reasonably priced supply of raw materials for the manufacturing of biodiesel. This reversible Eley–Rideal model response fits the experimental data with an R^2^ value of 0.989. At temperature ranges of 30–60 °C, the LHHW model with short interval assumptions produces an R^2^ value of 0.92–0.76. We were able to distinguish between the various functional groups present in biodiesel and Neat Diesel by using Fourier Transform Infrared Spectroscopy. When fatty acid methyl ester was discovered, the oil had been converted to biodiesel. Gas chromatography could identify both saturated and unsaturated fatty acids. This proves that biodiesel is a viable option for fuel and fuel-blending applications. Comparable to diesel’s calorific value of 44 MJ Kg^−1^, biodiesel’s calorific value was 32.25 MJ Kg^−1^. Therefore, for the best calorific value, it may be best to combine with ordinary diesel.

## Data Availability

The datasets used and/or analysed during the current study available from the corresponding author on reasonable request.
